# Quantifying the Monomer–Dimer Equilibrium of Tubulin with Mass Photometry

**DOI:** 10.1016/j.jmb.2020.10.013

**Published:** 2020-11-20

**Authors:** Adam Fineberg, Thomas Surrey, Philipp Kukura

**Affiliations:** 1 Physical and Theoretical Chemistry Laboratory, Department of Chemistry, University of Oxford, Oxford OX1 3QZ, UK; 2 The Francis Crick Institute, 1 Midland Road, London NW1 1AT, UK; 3 Centre for Genomic Regulation (CRG), Barcelona Institute of Science and Technology (BIST), Dr Aiguader 88, 08003 Barcelona, Spain; 4 ICREA, Passeig de Lluis Companys 23, 08010 Barcelona, Spain

**Keywords:** mass photometry, binding affinity, tubulin, single molecule, MP, mass photometry

## Abstract

The αβ-tubulin heterodimer is the fundamental building block of microtubules, making it central to several cellular processes. Despite the apparent simplicity of heterodimerisation, the associated energetics and kinetics remain disputed, largely due to experimental challenges associated with quantifying affinities in the <µM range. We use mass photometry to observe tubulin monomers and heterodimers in solution simultaneously, thereby quantifying the αβ-tubulin dissociation constant (8.48 ± 1.22 nM) and its tightening in the presence of GTP (3.69 ± 0.65 nM), at a dissociation rate >10^−2^ s^−1^. Our results demonstrate the capabilities of mass photometry for quantifying protein–protein interactions and clarify the energetics and kinetics of tubulin heterodimerisation.

## Highlights

• Quantifying high affinity protein–protein interactions is experimentally difficult.
• We use mass photometry to determine αβ-tubulin heterodimer energetics and kinetics.
• The *K*_d_ of the dimer is 8.48 ± 1.22 nM in the absence of added GTP.
• This lowers to 3.69 ± 0.65 nM upon GTP addition with a *k*_off_ > 10^−2^ s^−1^.
• Mass photometry is uniquely suited to study protein–protein interactions.

## Graphical abstract

**Figure:**
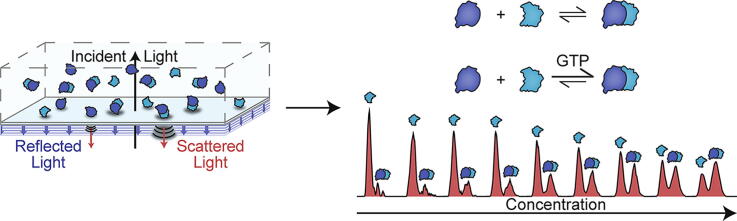

## Introduction

Microtubules, involved in processes as broad as mitosis, cell motility and intracellular transport, are constructed of heterodimers of α- and β- tubulin, highly conserved members of the tubulin/FtsZ family of proteins, with each subunit able to bind a GTP molecule.^1^ Heterodimer formation, the first critical step towards microtubule assembly, has been reported to require a number of cofactors.^2^, ^3^, ^4^ Studies on the thermodynamic and kinetic stability of αβ-tubulin heterodimers, which ultimately impacts the assembly and stability of microtubules, have produced a broad range of binding affinities and kinetics, ranging over 5 orders of magnitude from 10^−11^ to 10^−6^ M, with dissociation rates from 10^−5^ to 10^−2^ s^−1^.^5^, ^6^, ^7^, ^8^, [9] The variation in previous results can be attributed to a combination of various technical reasons and to biochemical differences between tubulins from different species.^10^ To achieve the required sensitivity, many single-molecule studies have had to rely on labelling and although care was taken to ensure that tubulin was not damaged by labelling, a quick, simple, label-free method is desirable because it excludes any potential perturbations.

**Figure 1:**
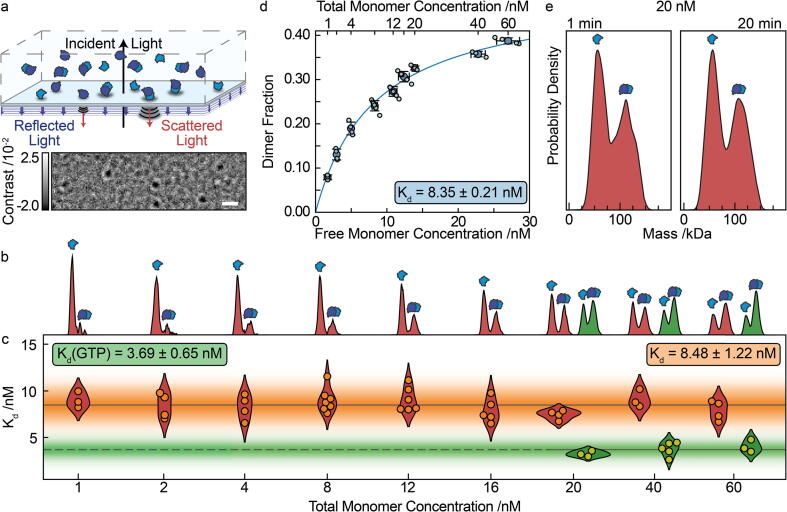
Quantification of tubulin heterodimersiation with mass photometry. (a) Schematic illustrating the operation of mass photometry. Imaging the interference between scattered and reflected light as a protein non-specifically binds at a glass-water interface results in label-free single molecule images with a contrast proportional to their molecular mass. Scale bar: 1 µm. (b) Mass kernel density estimates with 5 kDa bandwidth for tubulin at total monomer concentrations ranging from 1 to 60 nM without (red) and 20 to 60 nM with (green) GTP incubation. (c) Resulting binding affinities extracted from each distribution in (b). The grey lines indicate the global mean $K_{d}$ and the shaded areas the standard deviations for each data set. (d) Proportion of dimer present without GTP incubation at equilibrium as a function of free monomer concentration at equilibrium. Light blue markers indicate individual experiments, dark blue markers and error bars depict the mean and standard deviation respectively for each total monomer concentration. A logistic fit through the mean values yields $K_{d}$ = 8.35 ± 0.21 nM (error on fit). (e) Averaged mass kernel density estimates (*N* = 4) with 5 kDa bandwidth for tubulin without GTP incubation at a total monomer concentration of 20 nM from separate experiments 30 s after (left) and 20 min after dilution (right).

Experimental approaches capable of quantifying binding affinities in the sub-µM range in near-native conditions, however, are currently lacking. Label-free approaches generally require µM concentrations or higher, with higher dilutions only accessible through labelling, surface-based methods, or a combination of both. We have recently introduced mass photometry (MP), single molecule detection and mass measurement in solution based on light scattering.^11^ MP uses the interference between light scattered by a biomolecule as it non-specifically binds to a glass surface and the reflection of the illumination light from the glass-water interface to produce label-free images of single biomolecules (Figure 1(a)). The resulting optical contrast scales linearly with molecular mass, enabling the identification and counting of molecules and their complexes in solution. Detection is label-free, with the surface acting only as a detector and all interactions taking place in free solution between unmodified molecules over a concentration range from 0.1 to 100 nM, making MP in principle ideally suited to study tight protein–protein interactions in a quantitative fashion.^12^, ^13^, ^14^ Additionally, MP only requires a few tens of µl of sample for each measurement and takes less than a few minutes to run. This makes MP ideal for quickly determining thermodynamic and kinetic properties of protein interactions.

Applying MP to tubulin purified from porcine brain diluted at 60 nM concentration in BRB80 buffer exhibited a roughly 2:1 dimer:monomer ratio, in the absence of additional GTP, indicative of a low nM $K_{d}$. Accordingly, repeating these measurements at total tubulin concentrations ranging from 1 to 60 nM revealed a transition from predominantly monomeric towards predominantly dimeric distributions (Figure 1(b)). We can convert these distributions into binding affinities in multiple ways. Given that we are directly counting monomers and dimers, we can compute a $K_{d}$ from any one of these distributions given knowledge of the total protein concentration. The results are consistent across all concentrations measured yielding $K_{d}$ = 8.48 ± 1.22 nM (Figure 1(c)), in close agreement with a more traditional titration-based analysis, which requires multiple measurements to yield the affinity of interest (Figure 1(d)). We did not find significant differences between these measurements, performed after 20 min of equilibration after dilution, and those performed immediately after dilution (Figure 1(e)). During our measurements, which take 60–120 s, we could also not find any evidence of dissociation, suggesting that the associated off-rates must be faster than 10^−2^ s^−1^. Very slow dissociation rates would reveal identical monomer:dimer distributions for all dilutions because equilibrium would not be reached during the 20 min between dilution and measurement, and thus reflect the pre-dilution distribution. Incubation of the diluted and equilibrated tubulin with 1 mM GTP, by contrast, resulted in a clear shift towards dimer for a given total monomer concentration (Figure 1(c)) and an associated $K_{d}$ = 3.69 ± 0.65 nM. Due to the decrease in $K_{d}$ upon GTP addition, achieving an accurate measurement at low concentrations became challenging due to low statistics in the monomer population. However, due to the close agreement of measured $K_{d}$ values, without additional GTP, between the binding curve (Figure 1(d)) and the single shot (Figure 1(c)) measurements, we are confident in the accuracy of single shot measurements with MP.

These results quantify the binding affinity for the tubulin heterodimer both in the presence and absence of additional GTP and provide an upper limit to the dissociation rate, which is orders of magnitude faster than reports based on surface plasmon resonance.^8^ The resulting $K_{d}$ values, measured here for porcine brain tubulin, closely match those reported for rat brain tubulin in the absence of GTP (2.8 nM) in recent analytical ultracentrifugation experiments.^10^ By measuring dissociation of tubulin from different species and tissues, they report $K_{d}$ values ranging from 0.33 nM for chicken red blood cell tubulin to 47 nM for HeLa cell tubulin. This suggests that a $K_{d}$ of 3–10 nM may apply generally to mammalian brain tubulin. The reduction of the $K_{d}$ upon addition of GTP demonstrates the stabilising effect the nucleotide has upon the dimer, which considering the fast off rate suggests improved resistance to local fluctuations in tubulin concentration. This small change in binding affinity corresponds to a ΔΔG ≈ 2 kJ mol^−1^, highlighting the ability of MP to detect and precisely quantify even very subtle changes in protein–protein interactions, which opens the door towards investigating the effects of post-translational modifications. Moreover, the $K_{d}$ being on the order of nM demonstrates that, under physiological GTP and tubulin concentrations, tubulin will almost exclusively be found as a heterodimer, an important consideration as free β-tubulin is known to be toxic.^15^

The question remains: in what nucleotide state is the tubulin we measure without added GTP? The binding affinity of GTP to β-tubulin is on the order of a few 10s of nM,^16^, ^17^, ^18^ with GDP being bound less tightly, and the dissociation rate of GDP from the exchangeable site has a reported lower limit of 0.14 s^−1^.^19^, ^20^, ^21^ The purification method leaves tubulin with GTP bound at the non-exchangeable site and GDP at the exchangeable site and since the storage buffer contains no GTP, the bound GDP concentration will be on a similar order to the tubulin concentration. Under these conditions, therefore, if a significant concentration of β-tubulin had bound GTP, we would expect to see the measured $K_{d}$ increase as we dilute our tubulin sample, on our measurement timescales. However, we observe a zero gradient in $K_{d}$ across our dilutions in the absence of additional GTP, and a distinct lowering of the $K_{d}$ upon GTP addition. Additionally, α-tubulin has been thought to bind GTP many orders of magnitude stronger than β-tubulin,^6^ although the measurement was carried out at 0.67 µM tubulin concentration based on the assumption that at this concentration the dimer dissociated. As more recent $K_{d}$ measurements show, tubulin at this concentration will still be primarily dimeric, the nucleotide will be buried, and the $K_{d}$ low. It is possible that upon tubulin heterodimer dissociation the nucleotide at the α-tubulin can exchange more freely.

These observations would suggest that the observed decrease in $K_{d}$ upon GTP addition may result from nucleotide binding to β-tubulin, α-tubulin, or a combination of both, as compared to nucleotide being unbound at these sites at the low tubulin concentrations used in our experiments in the absence of additional GTP. Despite our use of HPLC purified GTP there is a possibility of trace amounts of GDP still being present, and therefore until the role of GDP binding upon tubulin dimerisation is further understood our results indicate a change in the apparent $K_{d}$ upon GTP addition. Given our results, it is tempting to speculate that the $K_{d}$ for tubulin heterodimer dissociation may mostly be affected by the presence of GTP at the non-exchangeable site that is directly located at the interface between α- and β- tubulin, whereas GTP at the exchangeable site that is situated between tubulin heterodimers controls the stability of their interaction during microtubule polymerization. Fortunately, MP is perfectly situated as a sensitive, label-free technique to further explore which of the two sites plays a larger role in tubulin dimer stability in the future.

## CRediT authorship contribution statement

**Adam Fineberg:** Formal analysis, Methodology, Software, Validation, Visualization, Writing - original draft, Writing - review & editing. **Thomas Surrey:** Resources, Writing - review & editing. **Philipp Kukura:** Conceptualization, Funding acquisition, Methodology, Supervision, Visualization, Writing - original draft, Writing - review & editing.

## Declaration of Competing Interest

Philipp Kukura is a founder, director and shareholder in Refeyn Ltd. Adam Fineberg and Thomas Surrey declare that they have no known competing financial interests or personal relationships that could have appeared to influence the work reported in this paper.
